# Green Radish Polysaccharides Ameliorate Hyperlipidemia in High-Fat-Diet-Induced Mice via Short-Chain Fatty Acids Production and Gut Microbiota Regulation

**DOI:** 10.3390/foods13244113

**Published:** 2024-12-19

**Authors:** Xiong Geng, Weina Tian, Miaomiao Zhuang, Huayan Shang, Ziyi Gong, Jianrong Li

**Affiliations:** Department of Food Science and Engineering, College of Food Science and Engineering, Bohai University, Jinzhou 121013, China; 18748135846@163.com (X.G.); kjcy10@163.com (W.T.); zmm13865981106@163.com (M.Z.); shy2023015150@bhu.edu.con (H.S.); xiaoxiong314@163.com (Z.G.)

**Keywords:** green radish, hyperlipidemia, polysaccharides, intestinal flora

## Abstract

The objective of this study was to examine the hypolipidemic effect and potential mechanism of action of green radish polysaccharide (GRP) in hyperlipidemic mice. We found that in mice fed a high-fat diet, supplementing with GRP reduced body weight and liver index, significantly improved serum lipid levels and markers of liver damage, and mitigated oxidative stress and inflammation. Mechanistically, in these hyperlipidemic mice, the size of fat cells was reduced by GRP, and the abnormal accumulation of lipid droplets was reduced. We also found that GRP regulates the composition of the intestinal microbiota, including the ratio of *Firmicutes* to Mycobacteria F/B and the levels of *Blautia* spp., which have been shown to alleviate liver damage and treat hyperlipidemia. Metabolite pathway analysis using the Kyoto Encyclopedia of Genes and Genomes identified the glycolysis/glycolytic metabolism and propionate metabolism pathways as potential targets for GRP in the amelioration of hyperlipidemia.

## 1. Introduction

Hyperlipidemia represents a significant contributor to the development of atherosclerosis. It is also a significant risk factor for hypertension and diabetes mellitus [1,2,3]. Hyperlipidemia is a condition characterized by abnormal blood lipid levels, specifically presenting as elevated levels of total cholesterol (TC), triglyceride (TG), and low-density lipoprotein cholesterol (LDL-C), in addition to reduced levels of high-density lipoprotein cholesterol (HDL-C). These changes are often accompanied by the presence of fatty liver and obesity [4]. From a biochemical perspective, a high-fat diet can induce the body’s inflammatory response [5] and increase the production of reactive oxygen species [6].

At present, hyperlipidemia is mainly treated by diet or medication, among which statins are the most widely used lipid-lowering drugs; however, rebound is frequently observed after drug treatment is stopped [7]. In addition, despite the use of these therapies for over 40 years, multiple adverse effects, such as an increased risk of diabetes, myalgia, hepatotoxicity, nephrotoxicity, and neurotoxicity, cannot be neglected. Importantly, due to varied responses to the applied therapies, many patients are insensitive to statins or unable to use them [8].

Considering the possible complications of hyperlipidemia and the side effects of conventional therapies, hyperlipidemic patients often introduce herbal products as an alternative or as an additional therapy [9]. Herbs are generally considered safe and associated with only minor side effects and are, additionally, recommended because of their availability, lower cost, and environmental benefits [10]. Clinical trials have identified the effectiveness of garlic, turmeric, artichoke, green tea, grape, Chios mastic gum, red yeast rice, and olive leaf in the regulation of one or more of the hyperlipidemia parameters [11]. Some common mechanisms of action of these herbs include the inhibition of lipid biosynthesis through the inhibition of HMG-CoA reductase [8,12], as well as the inhibition of other enzymes of cholesterol biosynthesis and lipolysis [13,14]. These herbs also control the lipid levels in the blood through the regulation of the lipid absorption process, as well as lipid and cholesterol excretion [15,16,17,18].

A multitude of naturally derived polysaccharides have been demonstrated to exert therapeutic effects on hyperlipidemia, though a comprehensive understanding of these effects remains elusive. For example, a study by Guo et al. [19] found that treatment with polysaccharides from the fungus *Grifola frondosa* prevented hyperglycemia and hyperlipidemia in diabetic mice. The underlying mechanism of this effect involved both changes to the bacteria that make up the gut microbiota and alterations to the expression of genes that regulate glycolipid metabolism in the liver [19]. Similarly, Hu et al. [20] demonstrated that polysaccharides derived from the flowering plant *Cyclocarya paliurus* possess a lipid-lowering effect. In this case, the mechanism of action involves the modulation of hepatic lipid metabolism-related enzymes, resulting in the alleviation of insulin resistance [20].

The biological activity of polysaccharides is contingent on a range of intrinsic characteristics. Such characteristics include the molecular weight (MW), monosaccharide composition, type of glycosidic bonds, and sulfate content [21]. The mechanism by which lipid-lowering is achieved is complex, in that it involves multiple targets and cell signaling pathways. In addition, the actual lipid-lowering effects tend to be inconsistent. Despite these complications, considerable effort has been invested in the development of new natural food sources of polysaccharides designed to mitigate hyperlipidemia in order to lower the incidence and impacts of cardiovascular and cerebrovascular diseases and to reduce related medical and societal costs.

One potential source of bioactive polysaccharides is the radish (*Raphanus sativus* L.), a cruciferous herb that is safe for consumption by infants as young as one year old. In the 2020 report of the Food and Agriculture Organization of the United Nations (FAO), the total planting area and output of radish and carrot were ranked first among vegetables [22]. The root of the radish is fleshy, with a spherical, conical, or oblong shape, and the root bark is white, red, green, purple, or pink [23]. With dark green leaves and lime green roots, green radish, also known as fruit radish, can be consumed raw or can be dried, made into soup, salted, or pickled. Green radish is juicy with a thin skin and fine flesh, a high sugar content, and a crunchy and tender texture, making it popular among the public [24,25]. Green radish has a long history of cultivation due to its nutrient density and its medicinal value as an expectorant, diuretic, and antidiarrheal agent [26,27,28].

Polysaccharides isolated from white radish, heart radish, and carrot have been shown to exert a variety of bioactive effects, including anti-obesity and anti-tumor metastasis effects, regulation of intestinal flora and hypoglycemia, and anti-inflammation, antioxidant, anticancer, and hepatoprotective effects [29]. These effects suggest that polysaccharides from green radish (GRP) might also exert similar activities. However, the activity of GRP in this context has not yet been explored. Therefore, in this study, we aimed to investigate GRP as a potential new functional food-related treatment for the alleviation of hyperlipidemia.

## 2. Materials and Methods

### 2.1. Preparation of GRP

Green radishes were purchased from Jinzhou General Supermarket (Liaoning Province, China). GRP was prepared from these radishes according to a previously reported procedure that uses an enzyme-assisted extraction [30]. Briefly, green radish samples were cleaned, dried at 30 °C to a constant weight, crushed, and sieved (40 mesh). To an accurately weighed sample of approximately 5 g of the dried material, 5 volumes of anhydrous ethanol were added, and the sample was placed in a bath at 50 °C for 8 h to remove the pigmentation. The resulting supernatant was filtered through gauze and discarded. The remaining residue was mixed with distilled water at a solid–liquid ratio of 1:21. A solution of 0.6% cellulase was added, and the pH value was adjusted to 7.0. The mixture was incubated at 48 °C for 59 min, and the enzyme was inactivated by treatment at 95 °C for 10 min. The extract was then subjected to vacuum filtration and alcohol precipitation, and the precipitate was collected by centrifugation. The precipitate was mixed with water and subjected to another round of centrifugation to obtain the supernatant. Finally, the mixture was freeze-dried to obtain crude GRP.

Protein was removed using the Sevage method. An aqueous solution of crude polysaccharide was prepared at a concentration of 1 mg/mL, and n-butanol and trichloromethane were added to this solution at a ratio of 1:5:25. The mixture was agitated for 20 min and then centrifuged for 15 min at 4500 rpm to remove the supernatant. This process was repeated at least three times until no protein layer remained. The supernatant was centrifuged and concentrated, followed by alcohol precipitation and freeze-drying to obtain GRP [31]. The characteristic polysaccharide absorption peak at 280 nm was confirmed using an ultraviolet (UV) spectrophotometer, and the GRP product was analyzed using Fourier-transform infrared (FT-IR) spectrometry over the wavelength range of 4000 to 500 cm^−1^ [32].

### 2.2. Animal Treatment and Experimental Design

All animal experiments were approved by the Animal Care and Use Committee at Jinzhou Medical University and followed the guidelines set by the Animal Research Advisory Committee. Four-week-old male Kunming mice (Jinzhou Medical University) were kept in groups of 5 per cage. The cage dimensions were 367 × 155 × 130. The mice were maintained under controlled lighting conditions (12 h light/dark cycle) at a relative humidity of 45 to 50% and a constant temperature of 20 to 22 °C. Autoclaved water and a commercial diet were provided ad libitum.

Each of the five mice were placed in a cage and kept in captivity. Thirty mice were divided into the normal control group (low-fat diet, LFD), GRP group, and high-fat model group (high-fat diet, HFD), with 10 mice in each group. Piglets in the normal control group were fed a basal diet, and those in the other two groups were fed a high-fat diet. In the GRP group, polysaccharide samples were mixed with a high-fat diet, and the polysaccharide dose was 200 mg/kg/d. The experiment period was 8 weeks. Both standard feed and high-fat feed were purchased from Jiangsu SyiePharma Bioengineering Co., Ltd. (Nanjing, China). The high-fat feed was stored in the refrigerator at −30 °C. The total caloric value of the basal diet was 3.83 kcal/g, and that of the high-fat diet was 4.46 kcal/g (protein 20.12%, fat 40.14%, carbohydrate 39.74%).

Following the treatment, fresh feces were proactively collected from 6 mice in each group. The stool samples were snap-frozen in liquid nitrogen and stored at −80 °C prior to 16S rRNA sequencing and quantification of short-chain fatty acids (SCFAs), including propionate and butyrate. After the treatment, all mice were sacrificed using 0.12 mg/kg isoflurane. Blood was collected from the eyeball of each mouse. This blood was incubated at room temperature for 2 h. Following centrifugation at 3000 rpm and 4 °C for 15 min, the serum was collected and frozen at −80 °C for subsequent analysis. The heart, liver, kidney, intestine, and epididymis fat were removed and rinsed extensively with PBS to remove blood and other debris, and the masses of the organs were determined.

### 2.3. Histopathological Analysis

The fixed tissue samples were dehydrated by immersion in a gradient of alcohol and xylene solutions. The tissues were embedded in paraffin, and sections of 5 μm were prepared and transferred onto glass slides. Liver, epididymal adipocytes, and colon tissue were promptly fixed with 4% paraformaldehyde before undergoing hematoxylin–eosin (HE) staining, and liver and epididymal adipocyte samples were subjected to oil red O staining. All stained tissue samples were observed and photographed under an optical microscope in order to identify microscopic changes to the tissues.

### 2.4. Serum and Liver Index Biochemical Analysis

Serum biochemical parameters, including triglycerides (TGs), total cholesterol (TC), low-density lipoprotein cholesterol (LDL-C), high-density lipoprotein cholesterol (HDL-C), aspartate aminotransferase (AST), and alanine aminotransferase (ALT), were determined using the appropriate commercially available kits (Nanjing Jianjian Co., Ltd., Nanjing, China) according to the manufacturer’s protocols. Tumor necrosis factor-α (TNF-α), interleukin-6 (IL-6), and lipopolysaccharide (LPS) were measured using commercially available kits (Nanjing Jianjian Bioengineering Research Institute, Nanjing, China) according to the manufacturer’s protocols; absorbances (λ = 405 nm) were determined using a plate reader.

In order to determine liver indexes, samples (0.2 g) of liver tissue were suspended in 9 volumes of 0.86% saline and then homogenized, and the supernatants were obtained by centrifugation at 3000 rpm for 15 min at 4 °C. The activities of the relevant liver enzymes (superoxide dismutase (SOD), catalase (CAT), and glutathione transferase (GSH-Px)) and the level of malondialdehyde (MDA) in the soluble fraction were determined using commercially available kits (Nanjing Jianjian Bioengineering Institute, Nanjing, China) according to the manufacturer’s instructions.

The validation parameters for the markers assessed were as follows: for TC, 0~19.39 mmol/L; for TG, 0.3~11.4 mmol/L; for LDL-C, 0.61~18.0 mmol/L; for HDL-C, 1.04~1.55 mmol/L; for AST, 2 to 100 U/L; for ALT, 0.156–10 mU/mL; for IL-6, 3~120 pg/mL; for LPS, 10–320 ng/L; for TNF-α, 25–800 ng/L; for SOD, 269.274 ± 23.448 U/mgprot; for CAT, 15.6–1000 pg/mL; for GSH-Px, 0.006–0.28U/mL; for MDA, 8.26 ± 2.75 nmol/mgprot.

### 2.5. High-Throughput Sequencing Analysis of Intestinal Microbiota

Total bacterial genomic DNA was extracted from the feces collected from all groups by using the GHFDE100 DNA isolation kit (Zhejiang Hangzhou Equipment Preparation 20190952, GUHE Laboratories, Hangzhou, China). The quality of DNA in a total of 108 samples was analyzed using a NanoDrop ND-1000 spectrophotometer (Thermo Fisher Scientific, Waltham, MA, USA) for quantification and by agarose gel electrophoresis for visualization.

Polymerase chain reaction (PCR) amplification of the bacterial gene encoding the V4 region of 16S rRNA was conducted using the forward primer 515F (5′-GTG CCA GCM GCC GCG GTA A-3′) and the reverse primer 806R (5′-GGA CTA CHV GGG TWT CTA AT-3′). Purification of the amplicons was achieved through the use of Agencourt AMPure XP Beads (Beckman Coulter, Indianapolis, IN, USA), and subsequent quantification was performed using the PicoGreen dsDNA Assay Kit (Invitrogen, Carlsbad, CA, USA). The amplicons were pooled in equal amounts, and paired-end 2 × 150 bp sequencing was conducted using the Illumina NovaSeq 6000 platform (GUHE Info Technology Co., Ltd., Hangzhou, China) [33].

The majority of the sequencing data were subjected to analysis using QIIME2 and relevant R packages (v3.2.0). The α-diversity indexes were calculated at the operational taxonomic unit (OTU) level using the OTU table that was generated by QIIME2. To assess the variations in microbial community structures among samples, a β-diversity analysis was conducted utilizing UniFrac distance metrics [34]. The outcomes were assessed through the implementation of principal coordinates analysis and non-metric multidimensional scaling [35].

The Kruskal test, which is accessible within the R stats package 4.4.1 (specifically the metagenomeSeq package), was employed to assess the taxonomic abundances at various levels, including genus, class, phylum, order, species, and family, across the samples or groups. The LEfSe method, which employs linear discriminant analysis (LDA) effect size, was utilized with the default parameters in order to identify taxa that exhibited differential abundance between groups [36].

### 2.6. Fecal SCFA Measurement

To 0.1 g of a fecal sample, 500 μL of a saturated sodium chloride solution was added, and the mixture was shaken and mixed with a crusher. To this mixture, 20 μL of a 10% H_2_SO_4_ solution was added, and the contents were vigorously shaken and mixed in a mixer. Anhydrous ether (500 μL) was added, and the sample was mixed by shaking and then centrifuged at 14,000 rpm for 15 min. The supernatant was transferred to a new tube containing 0.25 g of anhydrous sodium sulfate and then centrifuged again at 14,000 rpm for 15 min. This supernatant was transferred to a gas phase flask for subsequent analysis [37].

The following conditions were employed for the GC-MS analysis: RTX-WAX^®^ column (length: 30 mm; inner diameter: 0.25 mm; pore size: 0.25 μm; Restek, PA, USA); inlet temperature, 250 °C; column temperature, 100 °C; ion source temperature, 220 °C; and interface temperature, 250 °C. The ramp program consisted of a gradual increase in temperature over a specified period. The sample injection volume was 1 μL, the carrier gas was helium, the split ratio was 10:1, the flow rate was 2 mL/min, and the solvent delay was 1.5 min. The Q3 scan acquisition range was employed from 1.5 to 9.5 min, with a mass range (*m*/*z*) of 20.0 to 300.0 [38]. For the MS analysis, the electron bombardment ionization source was operated in the SIM scanning mode at an electron energy of 70 eV [39].

### 2.7. Statistical Analysis

The quantitative results were analyzed using SPSS 19.0 and ImageJ V1.8.0. Statistical comparisons of multiple groups were conducted using an analysis of variance, with Dunnett multiple comparisons employed to assess group differences. Comparisons between two groups were conducted using the *t*-test. Statistical significance was defined as differences leading to *p*-values less than 0.05.

## 3. Results and Discussion

### 3.1. Characterization of GRP

The GRP was subjected to UV spectroscopy. No absorption peaks were observed at 260 and 280 nm (Figure S1). The GRP was characterized using FT-IR spectroscopy analysis. The strong absorption peak of green radish polysaccharide (GRP) at 3440.81 cm^−1^ is attributed to the O–H stretching vibration, whereas the peak observed at 2927.04 cm^−1^ is ascribed to the C–H stretching vibration. The peak at 1633.3 cm^−1^ is attributed to the C=O vibration of the ester carbonyl group, whereas the peak near 1016.56 cm^−1^ indicates the presence of a glucopyranose ring in GRP. These peaks are characteristic of sugars. Additionally, the peak at 599.08 cm^−1^ is attributed to the O–H surface bending vibration (Figure S2).

### 3.2. Effects of GRP on Body Weight and Organ Index of HFD-Fed Mice

We assessed the effects of GRP on hyperlipidemia using a mouse model, in which control mice fed LFD were compared to mice fed HFD and to mice fed HFD that included GRP. Following the 8-week treatment period, HFD-fed mice had significantly higher body weights than the LDF-fed mice (*p* < 0.05). However, mice fed HFD with GRP had significantly reduced body weights in comparison with mice fed unsupplemented HFD (*p* < 0.05).

**Figure 1:**
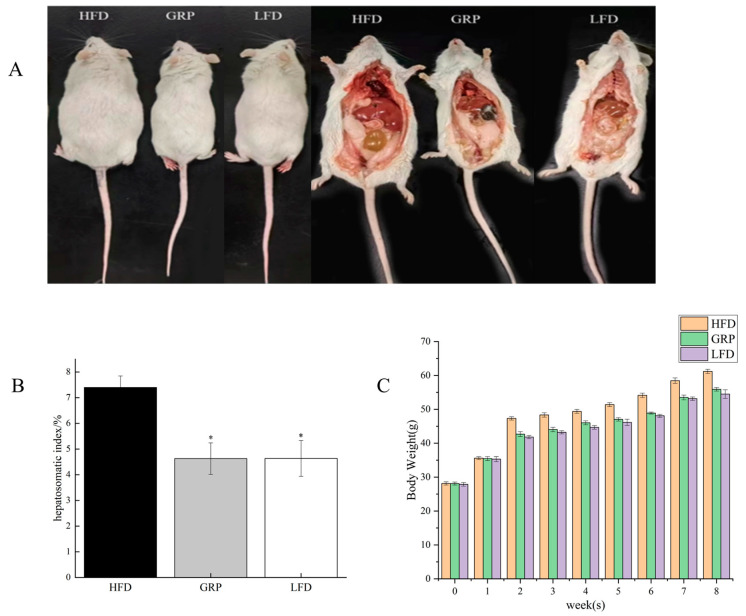
GRP reduced HFD-induced body weight gain; (**A**) representative pictures of mice; (**B**) hepatosomatic index of mice; (**C**) diagram of changes in mice body weight. * *p* < 0.05.

In addition to measuring body weights, we also determined the hepatosomatic index (HSI) of the mice after the treatment period. HSI is typically used to evaluate the function and morphology of the liver in order to study the progress of a disease or for other similar reasons. The liver is a key metabolic organ that can metabolize and remove toxins and waste products from the body; it is also the center of the synthesis of some other important substances that are used throughout the body [40]. By measuring the HSI, the function of the liver can be assessed, and if the index is abnormal, it may indicate poor liver function or the presence of disease [41]. The assessment of HSI facilitates the elucidation of pathological alterations in liver function, and it offers invaluable insights for the advancement of therapeutic approaches and the prevention of related ailments. Here, we observed a significant difference in the HIS between LFD- and HFD-fed mice (*p* < 0.05), and we observed a significant difference in the HIS between mice fed HFD alone and mice fed HFD with GRP (*p* < 0.05). Thus, this analysis revealed that HFD-dependent weight gain and liver changes were mitigated by the inclusion of GRP in the diet (Figure 1C), indicating that GRP inhibits weight gain and suggesting that it impacts the liver in a positive way.

### 3.3. Effects of GRP on the Serum Lipid Levels of HFD-Fed Mice

**Figure 2:**
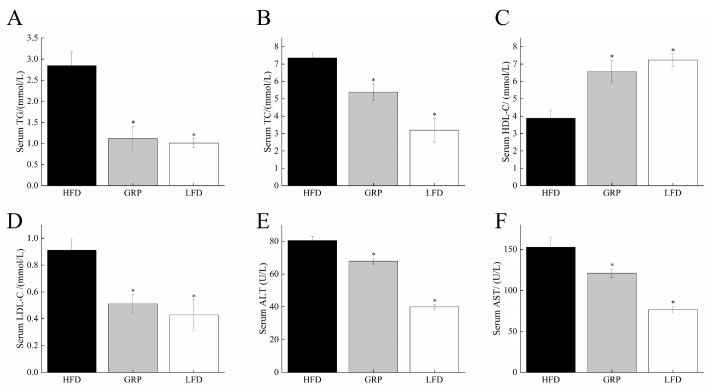
Effects of GRP on serum biochemical indicators. The levels of TG, TC, HDL-C, AST, ALT, and LDL-C in the serum were evaluated (**A**–**F**). The results are expressed as means ± SD. Statistically significant results between all groups were expressed by lowercase letters. * *p* < 0.05.

We next determined serum levels of lipid-related molecules and liver-related enzymes in the three groups of mice. As shown in Figure 2, as compared with the LFD group, the serum levels of TG, TC, and LDL-C and the serum AST and ALT activities were significantly increased in the HFD group (all *p* < 0.05), indicating that the hyperlipidemia mouse model was successfully established. These differences were consistent with a model in which the increase in fat mass in HFD mice resulted in lipid metabolism disorders [42]. Importantly, we also observed significant differences in these parameters between the HFD group and the group fed HFD with GRP. Specifically, in the GRP group, the TG, TC, and LDL-C levels were found to be significantly lower than those in the HFD group (all *p* < 0.05), while the HDL-C levels were significantly higher in mice fed HFD with GRP (*p* < 0.05).

Lipids are insoluble in water, necessitating their association with lipoproteins to facilitate transport in aqueous solutions [43]. These complexes include LDL-C and HDL-C [44]. It has been demonstrated that HDL acts as a carrier for cholesterol from peripheral tissues to the liver, facilitating the subsequent breakdown of cholesterol [45]. Accordingly, higher levels of HDL are associated with a reduced risk of cardiovascular disease [46]. Therefore, our results, which suggest decreased LDL-C and increased HDL-C, indicated that GRP was associated with improvements in blood lipid levels in hyperlipidemic mice.

The serum ALT and AST levels have long been regarded as markers of liver damage, encompassing a diverse array of etiologies including viral hepatitis and non-alcoholic fatty liver disease [47], and these enzymes are the most sensitive indicators of hepatocellular injury. Aminotransferases have been demonstrated to be strong predictors of metabolic syndrome and cardiovascular disease [48], and the overall risk of metabolic disease may also be influenced by these factors. This evidence supports the hypothesis that obesity, type 2 diabetes, atherosclerosis, and thrombosis risk profile are all associated with an individual’s aminotransferase profile [48]. Consequently, it is reasonable to posit that aminotransferases may be regarded as surrogate biomarkers of hepatic metabolic function, transcending classical notions of hepatocyte injury [48]. Indeed, the enzymatic activity observed in aminotransferases may reflect key aspects of liver function at the level of physiology and pathophysiology [48]. In the present study, we found that the serum ALT and AST levels were significantly increased by HFD (*p* < 0.05), but the inclusion of GRP with the HFD was associated with a significant reduction relative to HFD alone (*p* < 0.05), indicating that GRP may be an effective intervention for mitigating liver damage associated with hyperlipidemia.

### 3.4. Effects of GRP on Oxidative Levels and Inflammatory Indicators in HFD-Fed Mice

**Figure 3:**
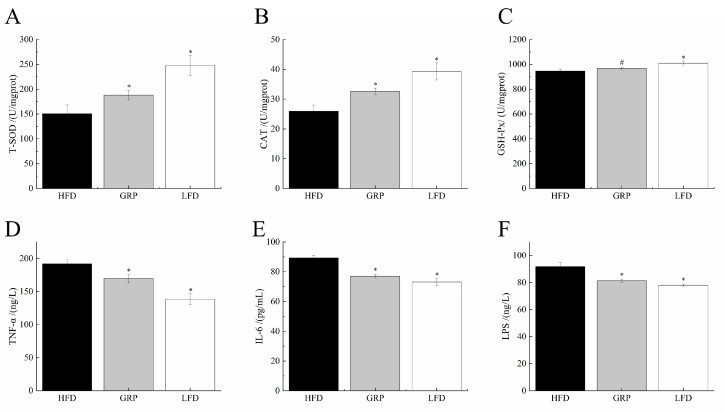
Effects of GRP on oxidative levels and inflammatory indicators. The levels of SOD, CAT, GSH-Px, TNF-α, IL-6, and LPS were evaluated (**A**–**F**). The results are expressed as means ± SD. Statistically significant results between all groups were expressed by lowercase letters. * *p* < 0.05 and ns: no significance.

Oxidative stress involves the excessive production of reactive oxygen species (ROS), including oxygen, and can occur when an organism is subjected to a variety of noxious stimuli [49]. Specifically, oxidative stress occurs when the degree of oxidation exceeds the removal of oxides, leading to an imbalance between the oxidative and antioxidant systems and, consequently, tissue damage [49]. This imbalance has been identified as one of the key factors contributing to the development of diseases associated with an elevated level of lipids in the bloodstream, including atherosclerosis, coronary artery disease, and stroke [50]. The presence of excessive quantities of free radicals in the body, particularly those containing oxygen atoms, can cause damage to tissues and cells [51]. These radicals may also initiate a process known as lipid peroxidation, which is the deterioration of lipids. These radicals can also disrupt lipid metabolism [52]. GRP has been observed to enhance the antioxidant capabilities of the human body while concomitantly reducing the production of free radicals. The mechanisms underlying these effects involve stimulating the activity of antioxidant enzymes, including SOD and CAT, and by enhancing the activity of GSH-Px. Collectively, these enzymes act to reduce the peroxidation of lipids in the serum (Figure 3A–C).

Hyperlipidemia is generally accompanied by low-grade chronic systemic inflammation. Levels of IL-6 have been shown to correlate closely with dyslipidemia, and this cytokine is known to play important roles in the regulation of lipids and body weight. The activity of IL-6 can be regulated by inhibiting TNF-α and by reducing insulin resistance and blood glucose and lipid levels [53]. Additionally, research has indicated that polysaccharides can limit inflammation [21]. Consequently, the inhibition of pro-inflammatory cytokine activation may represent an efficacious approach for the treatment of hyperlipidemia. In the mouse model, we found a significant reduction in the levels of IL-6 and TNF-α in the serum of mice fed an HFD with GRP in comparison with mice fed an unsupplemented HFD (*p* < 0.05). Based on this result, we hypothesized that the intestinal dysbiosis observed in the HFD group may have affected intestinal permeability, which in turn would lead to an increase in the influx of LPS of bacterial origin into the bloodstream. Indeed, mice fed HFD supplemented with the GRP group were found to have significantly reduced LPS in serum relative to HFD mice (*p* < 0.05). These results indicate that GRP has exerted antioxidant and anti-inflammatory effects in mice (Figure 3D–F).

### 3.5. Effects of GRP on Histopathology of Liver Epididymal Fat and Intestinal Tissue in HFD-Fed Mice

**Figure 4:**
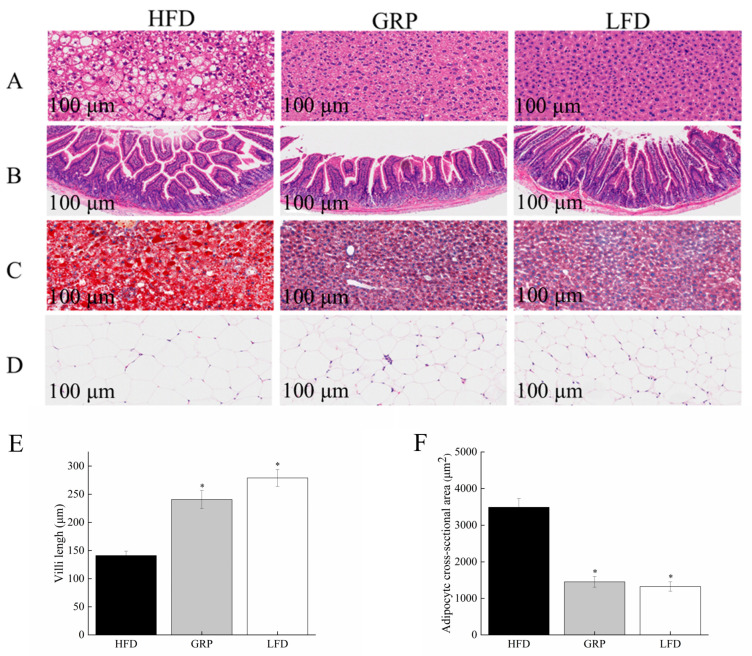
GRP ameliorates defects in gut, liver, and adipose tissue cells’ structure induced by high-fat diet. (**A**) Representative pictures of hematoxylin and eosin (H&E) staining for liver tissue. (**B**) Representative images of HE staining in the ileum of mice were shown. (**C**) Representative pictures of oil red O staining for liver fat. (**D**) Representative pictures of hematoxylin and eosin (H&E) staining for adipose tissue. Pictures were shown as 20× zoom, scale: 100 μm. (**E**) Change in the colon villi length of the mice in the three groups. (**F**) Mean size of mice adipocytes. Statistically significant results between groups are indicated by small letters. * *p* < 0.05.

Figure 4 depicts representative histological images of sections of liver isolated from each of the experimental groups. The hepatocytes in samples from the LFD group were found to be arranged in an orderly fashion, exhibiting intact cell membranes and a distinct morphology so that hepatocytes could be identified unambiguously. By contrast, samples from HFD mice (Figure 4A) displayed many lipid droplet vacuoles, accompanied by the appearance of numerous fat vacuoles [54]. These intracellular changes were accompanied by a notable enlargement of the intercellular space and a correspondingly disorganized arrangement of cells within that space. In samples from mice also administered GRP, however, the morphology of hepatocytes was markedly improved, with a notable reduction in lipid droplets, inflammatory cells, and intercellular space. In sections subjected to oil red O staining (Figure 4C), samples from the HFD group exhibited an accumulation of lipid droplets, suggesting a disruption of lipid metabolism in the cells, but the number of lipid droplets in samples from the GRP group was reduced. Taken together, these results demonstrate that GRP reduces the abnormal accumulation of lipid droplets and restores normal hepatocyte morphology.

As illustrated in Figure 4B,E, the intestinal mucosa of the mice in the LFD group displayed a distinct structure, with intact villi and a regular distribution of glands. A comparison of the intestinal tracts of mice in the LFD and HFD groups revealed notable structural differences. For example, the villi in the intestines of mice in the HFD group were considerably shorter than those in the LFD group. However, in HFD mice treated with GRP, the length of the villi in the gut increased.

Figure 4D depicts representative images of epididymal adipose tissue sections derived from the experimental groups. The cells of the adipose tissue isolated from mice in the HFD group were markedly increased in size relative to those from the LDF group, but this effect of the HFD was mitigated by GRP. Upon size quantification (Figure 4F), HFD mice were found to have significantly larger adipocytes relative to LFD mice (*p* < 0.05), whereas HFD mice treated with GRP were found to have significantly smaller adipocytes than HFD mice (*p* < 0.05).

### 3.6. Effect of GRP on Intestinal Microbial Structure in HFD-Fed Mice

**Figure 5:**
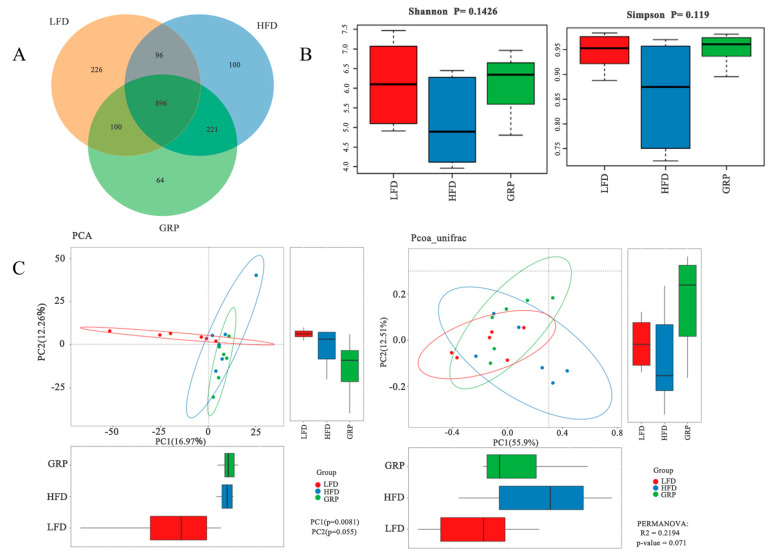
Effect of GRP on intestinal microbial structure in HFD-fed mice. (**A**) Statistical differences between the groups of alpha diversity; (**B**) Venn diagram of the ASVs for LFD, HFD, and GRP; (**C**) β-diversity PCA and PCoA results.

As the microbiome is well known to influence host metabolism, we next investigated changes to gut microbes in LFD mice and in control or GRP-treated HFD mice. As illustrated in Figure 5A, the number of OTUs observed at the 97% sequence similarity level was 3912. Of these, a total of 1318 were identified in the LFD group; 1313 in the HFD group; 1281 in the GRP group; 896 in the LFD, HFD, and GRP groups; 96 in the LFD and HFD groups; 100 in the LFD and GRP groups; and 221 in the HFD and GRP groups. Alpha diversity is frequently utilized as a metric to quantify species diversity and uniformity. As demonstrated in Figure 5B, the Shannon and Simpson indices exhibited a markedly diminished value for the HFD group in comparison to the LFD and HFD/GRP groups. Conversely, the indices for the GRP group demonstrated a significantly higher value than those observed for the HFD group. A comparison of the Shannon and Simpson indices of the HFD and LFD groups revealed significantly lower values for the HFD group, while the index of the HFD/GRP group was significantly higher than that of the HFD group.

As shown in Figure 5C, the uniformity of the rodent species declined in HFD mice, while the intervention with GRP improved the diversity and uniformity of the intestinal microbiota of the HFD mice [55]. In order to better characterize the similarity of microbial composition between individuals, the measurement of gender was conducted in accordance with the principles of beta diversity. A clear differentiation was observed between the microbiota of the HFD and that of the LFD group, indicating that the high-fat diet changed the microbiota structure of the mice [56]. However, the distribution of microbiota in HFD mice supplemented with the GRP group was different from that of the HFD group.

### 3.7. Effect of GRP on Intestinal Microbial Composition in HFD-Fed Mice

**Figure 6:**
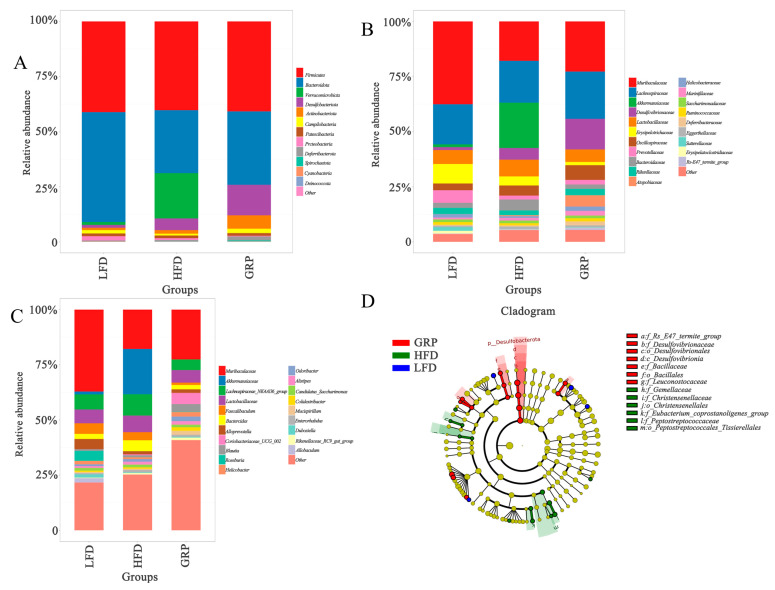
GRP changed the composition of gut microbiota in HFD-fed mice. (**A**) Average relative abundance at the phylum level in each group. (**B**) Average relative abundance at the family level in each group. (**C**) Average relative abundance at the genus level in each group. (**D**) LEfSe analysis was conducted to identify fecal microbial taxa that accounted for the greatest differences among all the groups.

At the level of the phylum (Figure 6A), the ratio of thick-walled and bacillomimetic phylum (F/B) is associated with the maintenance of homeostasis in vivo, and changes in this ratio may lead to a variety of pathologies, with the increased abundance of specific thick-walled phylum or bacillomimetic species, leading to obesity and intestinal inflammation [56]. A significant decrease in the relative abundance of bacteriophages was observed within the HFD group as compared to the other groups. It is noteworthy that the relative abundance of the thick-walled phylum remained largely unchanged in the HFD, GRP, and LFD groups. In contrast, the GRP intervention led to a reduction in the ratio of thick-walled to facultative phyla (F/B) and an increase in the relative abundance of *Actinobacteria*. These findings suggest that GRP may have contributed to the improvement in the intestinal flora of the hyperlipidemic mice, as the composition of the microbiota was adjusted toward that of the LFD group.

At the family level (Figure 6B), the GRP treatment was observed to increase the relative abundance of beneficial bacteria belonging to the *Lachnospiraceae*, *Oscillospiraceae*, *Marinifilaceae*, and *Ruminococcaceae* families, which is consistent with the trend of intestinal flora seen upon the use of a lyophilized powder of sand onion as a lipid-lowering agent in hyperlipidemic mice [57]. Authoritative studies have shown decreases in the abundance of families such as *Ruminococcaceae* and *Lachnospiraceae* in subjects with newly diagnosed or longstanding diabetes compared to normal glucose-tolerant subjects [57]. *Lachnospiraceae* and *Ruminococcaceae* hydrolyze starch and other sugars to produce butyrate and other SCFAs. Lachnospiraceae represent a central aspect of the intestinal microbiota, colonizing the intestinal lumen immediately following birth and subsequently increasing in species richness and relative abundance over the lifespan of the host [58]. Additionally, they are a significant contributor to the production of SCFAs, and genome analyses of *Lachnospiraceae* have revealed a considerable capacity to utilize polysaccharides from dietary sources [58]. The main fermentation metabolites of *Ruminococcaceae* are acetic acid and formic acid, and the abundance of this genus has been shown to be positively correlated with gut motility.

At the genus level (Figure 6C), GRP increased the relative abundance of *Odoribacter*, *Blautia,* and *Muribaculaceae*. Among them, *Blautia* is known to have a probiotic effect that can maintain intestinal homeostatic activity by preventing inflammation and promoting the production of SCFAs, strengthen the tight junctions of intestinal epithelial cells, and prevent pathogenic bacterial infections, and increased levels of *Blautia* are associated with an improved prognosis in colon cancer and cirrhosis of the liver [59]. In addition, *Blautia* intervention can reduce the levels of inflammatory factors (TNF-α, IL-1β, and IL-6), increase the content of SCFAs, regulate the structure of intestinal flora to alleviate inflammation, and reduce LPS-induced injury in the liver, lung, and colon by lowering the levels of liver injury markers (ALT and AST) and increasing the activities of antioxidant enzymes (SOD and GSH-Px) in liver tissues. It can reduce LPS-induced liver, lung, and colon injury, especially in acute liver injury [60], and *Muribaculaceae* can help to inhibit intestinal barrier dysfunction, inflammation, and lipid metabolism disorders [61].

Significant differences in microbiota among LFD, HFD, and GRP were observed, as shown in Figure 6D, where f_Christensenellaceae, f_Christensenellaceae, o_Peptostreptococcales_Tissierellales, and f_Eubacterium_coprostanoligenes_were significantly enriched in the HFD group. The o_Peptostreptococcales_Tissierellaceae was significantly increased in the HFD group, with o_Peptostreptococcales_Tissierellales negatively correlating with taurosterodeoxycholic acid (TADO). The f_Eubacterium_coprostanoligenes_group was observed to have significantly elevated concentrations of fumaric acid, malic acid, citric acid, oxoglutaric acid, adenine acid, and uric acid, in addition to cholesterol. These metabolites were found to have a positive correlation between the *Eubacterium coprostanoligenes* group and the aforementioned variable and were negatively correlated with L-glutamic acid, citrulline, glycine, and CDCA. The f_Eubacterium_coprostanoligenes_group is positively associated with atherosclerotic injury [62]. A negative correlation has been observed between L-lysine and f_Christensenellaceae [63]. Lysine is an alkaline amino acid. Microbiota-derived SCFAs play particularly important roles in the crypts and colon of the small intestine.

### 3.8. Effect of GRP on SFCAs in HFD-Fed Mice

**Figure 7:**
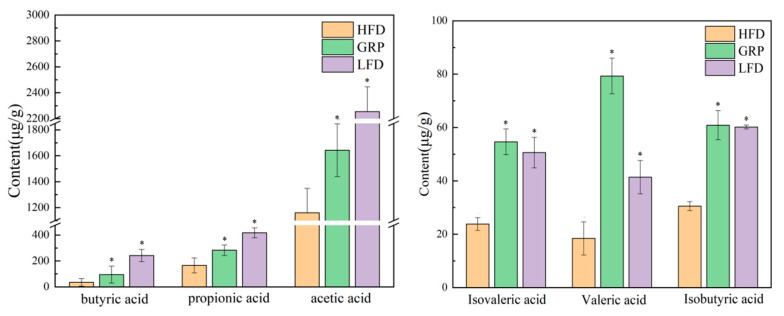
Contents of fecal short-chain fatty acids in three mice groups; statistically significant results between all groups were expressed by lowercase letters. * *p* < 0.05.

SFCAs promote the absorption of indigestible products in the body and the proliferation of colonic epithelial cells and mucosal growth and thus exert a profound influence on the health of the host [64]. Certain non-absorbent components in food can undergo metabolism to produce SCFAs, including acetate, propionate, and butyrate [64]. Here, we measured the levels of the following SCFAs in the guts of mice fed LFD, HFD, or HFD with GRP: butyric, propionic, acetic, valeric, isovaleric, and isobutyric acids. As illustrated in Figure 7, a significant reduction in the SCFA concentration was observed in the cecum of the HFD group in comparison with the LFD group (*p* < 0.05). However, we observed a significantly increased total level of SCFAs in the cecum of mice fed HFD with GRP as compared to mice fed HFD alone (*p* < 0.05).

Specifically, we found that intervention with GRP elevated butyric acid levels in the hyperlipidemic mice. Butyric acid, as one of the fermentation products of intestinal flora, not only acts as a source of energy to the microbes and the host but also acts as a signaling molecule that regulates the metabolic processes of the host. For example, several studies have shown that butyric acid acts as a substrate for fat synthesis on the one hand and as a signaling molecule on the other hand, and that it regulates fat metabolism through the activation of specific GPCRs and by inhibiting histone deacetylases [65,66]. The GRP intervention also significantly increased the levels of propionic acid and acetate (*p* < 0.05). Propionic acid has been observed to reduce the levels of fatty acids in the liver and plasma, to decrease food intake, and to exert an immunosuppressive effect while potentially improving tissue insulin sensitivity; these findings suggest that GRP may be beneficial in preventing obesity and type II diabetes [67].

Acetic acid reduces levels of multiple inflammatory factors, including TNF-α, IL-1β, and IL-6, which are responsive to LPS stimulation, while stimulating increases in the anti-inflammatory factor IL-10. Acetic acid also plays an important regulatory role in promoting adipocyte differentiation, suppressing the accumulation of adipose tissue, and alleviating obesity-induced insulin resistance. This outcome is achieved by regulating the transcription factors and signaling pathways that control gene expression [68].

GRP significantly increased the levels of two branched SCFAs, isobutyric and isovaleric acids, relative to the levels in HFD. In the mice fed HFD with GRP, the levels of these two SCFAs were comparable to those in LFD mice, suggesting that GRP strongly mitigates the increase in isobutyric and isovaleric acids observed in hyperlipidemia. Branched SCFAs, including isobutyric and isovaleric acids, are produced by the fermentation of branched-chain amino acids, which are produced from undigested proteins that reach the colon. Isobutyric and isovaleric acids affect adipocytes as well as lipid and glucose metabolism, and they help to improve insulin sensitivity in individuals with metabolic disorders [69].

GRP also led to a significant increase in valeric acid when administered with HFD. This SCFA exerts multiple health benefits by altering the metabolism of T- and B-lymphocytes and through immunomodulatory effects mediated by epigenetic reprogramming. Valproate, a derivative of valeric acid, has been observed to exert a beneficial effect on the immune response in mice, specifically in relation to the gut and brain. The observed amelioration was attributed to the drug’s ability to balance metabolic and epigenetic regulation and to promote anti-inflammatory immune responses [70]. Therefore, our finding that GRP mitigates decreases in valeric acid suggests a mechanism by which GRP intervention can have an anti-inflammatory effect.

### 3.9. Metabolite Pathway Analysis

An analysis of the signaling pathways affected by GRP supplementation was performed using KEGG, and a Metabolic Pathway Analysis (MetPA) was performed. These analyses identified five key metabolites involved in 13 metabolic pathways that differed between HFD mice and mice fed HFD supplemented with GRP. Specifically, the metabolic pathways that differed significantly between these two groups (*p* < 0.05) were propanoate metabolism, sulfur metabolism, glycosaminoglycan metabolism, sulfur metabolism, glycosaminoglycan biosynthesis–heparan sulfate/heparin, glycolysis or gluconeogenesis, cholinergic synapse, taurine and hypotaurine metabolism, butylated metabolites metabolism, butanoate metabolism, pyruvate metabolism, nicotinate and nicotinamide metabolism, and phosphonate and phosphinate metabolism. The effect of GRP on the propionate metabolism pathway was found to be highly significant (*p* < 0.001).

Hyperlipidemia represents a significant contributing factor to the development of lipid metabolism disorders, which in turn can lead to the onset of metabolic dysfunction–associated steatotic liver disease (MASLD) [71]. Elevated serum concentrations of TC, TG, and LDL-C are notable factors contributing both to the development of cardiovascular disease and to disturbances to the intestinal flora, which often correlate with gut barrier dysfunction [72]. In this study, in HFD mice, we found marked alterations to lipid profiles, as well as increases in key indicators of liver function, suggesting that the HFD mice were experiencing some degree of liver injury. Conversely, it has also been shown in humans that liver dysfunction resulting from liver injury or the accumulation of fatty substances within the organ predisposes individuals to an irregularity in lipid metabolism [73]. Importantly, we found that an intervention with GRP can mitigate hepatic injury and hepatic steatosis, which might facilitate hepatic lipogenesis, as noted by positive changes to the lipid profiles of the mice.

Modifications to dietary habits can influence the composition and characteristics of the intestinal microbiota, with potential implications for the development of disease or maintenance of health [74]. Consequently, we hypothesized that the obesity and hepatotoxicity observed in animals exhibiting hyperlipidemia may also be associated with shifts in the microbial composition [75]. Here, we present evidence that GRP has a significant impact on the ratio of Firmicutes and Bacteroidetes (F/B), with a notable increase in the relative abundance of beneficial bacteria Lachnospiraceae and Marinifilaceae at the family level, and an increase in the relative abundance of Blautia and Muribaculaceae at the genus level. Lachnospiraceae are a core component of the gut microbiota [59]. These bacteria colonize the intestinal lumen from the moment of birth, and their species diversity and relative abundance increase throughout the lifespan of the host [59]. The *Lachnospiraceae* family plays a key role in the production of SCFAs, which are important in maintaining gut health and function by providing energy for colonocytes.

Additionally, the use of GRP intervention was demonstrated to prevent weight gain and to enhance the concentrations of SCFAs within the cecum. It has been demonstrated in studies that SCFAs play an essential role in the prevention of obesity and in the promotion of blood lipid metabolism in hyperlipidemic mice [76]. Furthermore, SCFAs have been demonstrated to stimulate the expression of fatty acid oxidase, thus preventing the conversion of fatty acids to triglycerides by means of the upregulation of the activity of peroxisome proliferator-activated receptors (PPARs) [77]. This study demonstrated a significant enhancement in SCFA concentrations following the GRP intervention, accompanied by a notable expansion in the relative abundance of SCFA-producing Lachnospiraceae bacteria. It thus can be concluded that one mechanism underlying the anti-hyperlipidemic effect of the GRP intervention involves the upregulation of SCFA-producing microbes.

Acetate is an important product downstream of glycolysis, in which bacteria break down sugars, including polysaccharides or monosaccharides obtained from the hydrolysis of polysaccharides by other bacteria. In addition, acetate-producing bacteria directly convert carbon dioxide and reduced hydrogen into acetyl-CoA via the acetyl-CoA pathway [78]. A substantial body of research has indicated that supplementation with acetate, propionate, or butyrate can reduce hepatocellular lipid accumulation, attenuate hepatic inflammation, and inhibit cholesterol synthesis in animals [79]. This effect appears to occur through a mechanism involving an increase in hepatic lipid antioxidant levels, a decrease in TNF expression, an increase in fibroglycogen storage, and a decrease in hepatic fatty acid synthase activity through the AMPK acetyl-CoA carboxylase pathway [80]. This mechanism is consistent with the trends observed in the levels of hepatic antioxidants and TNF that we observed among the control, HFD, and HFD/GRP mice; however, further validation of the association with fatty acid synthase activity is needed.

The catabolic pathway of propionic acid begins with its conversion to propionyl-CoA. Since propionic acid has three carbon atoms, propionyl-CoA does not directly enter β-oxidation or citric acid cycles [81]. Instead, propionyl-CoA undergoes carboxylation to form D-methylmalonyl-CoA, which then undergoes isomerization to form L-methylmalonyl-CoA [82]. This latter intermediate is converted to succinyl-CoA by a vitamin B12-dependent enzyme, after which it proceeds to the citrate cycle. The citric acid cycle represents a central metabolic pathway, integrating carbohydrate, fat, and protein metabolism [83]. This coordination occurs through the linking of these three types of molecular substrates in the form of acetyl-CoA. Through the enzymatic reactions of the citric acid cycle, acetyl-CoA undergoes complete oxidation, resulting in the formation of carbon dioxide and water, releasing energy and providing a major source of energy for cells [84,85]. Thus, the potential pathway by which GRP exerts its lipid-lowering effect is through enriching the abundance of SFCA-producing bacteria, promoting the production of SCFAs, and converting the excess carbohydrates and fats into energy through the citric acid cycle to prevent them from accumulating into fat in the body. We found that the GRP intervention induced a notable increase in the number of SCFA-producing bacteria and the concentration of SCFAs in the intestine of HFD mice, which correlated with a significant anti-hyperlipidemic effect. In short, we found that glycolysis/glycolysis metabolic and propionate metabolic pathways are key targets of the action of GRP in ameliorating hyperlipidemia.

## 4. Conclusions

In this study, we used green radish as raw material to prepare polysaccharide and investigated the lipid-lowering effect of GRP. In animal experiments, GRP was shown to increase antioxidant levels, reduce the content of serum lipids, and inhibit the activation of pro-inflammatory factors, thereby improving the liver and intestinal damage caused by an HFD. It also reduced the volume of fat cells, further inhibiting weight gain. Both HFD and GRP were found to affect the number of intestinal microorganisms at all taxonomic levels. After intervention with GRP, the richness and diversity of the intestinal microbiota was restored, and the increased ratio of Firmicutes/Bacteroides in the intestinal microflora caused by HFD is reduced, while the relative abundance of beneficial bacteria such as Actinobacteria is increased. According to targeted metabolism, it was found that GRP can increase the content of SCFAs, indicating that the supplement can improve hyperlipidemia by enriching SCFA-producing bacteria, including *Blautia*.

Glycolysis/gluconogenesis metabolism and propionic acid metabolism pathways are potential targets of GRP to improve hyperlipidemia. In accordance with the metabolic processes and structural composition of the intestinal microbiota, it was inferred that the GRP intervention could achieve lipid-lowering by regulating the gut microbiota and metabolite production, increasing antioxidant levels, lowering serum lipid levels, and inhibiting the activation of pro-inflammatory factors, thus ameliorating liver and intestinal injuries induced by an HFD and further inhibiting weight gain.

On this basis, the limitations of this study and the next main direction of discussion are as follows. First, we recognize that glycolytic/gluconeogenic metabolism and propionic acid metabolism pathways are only potential targets, and the molecular mechanisms by which GRP might influence these pathways need to be further explored. Second, in order to improve the yield and decolorization of the polysaccharides during preparation, the material was heated to 50 °C. Further research will need to be performed to determine whether this heating reduced biological activity and to optimize the preparation of GRP. Finally, future studies should assess the effects of different doses of GRP to elucidate other metabolic responses along the gut–hepatic axis and related pathways.

## Data Availability

The original contributions presented in this study are included in the article/Supplementary Materials. Further inquiries can be directed to the corresponding author.

## Supplementary Materials

The following supporting information can be downloaded at: https://www.mdpi.com/article/10.3390/foods13244113/s1, Figure S1. UV absorption spectrum of GRP; Figure S2. GRP IR spectrum.
