# Supplementary figures and images for: Green Radish Polysaccharides Ameliorate Hyperlipidemia in High-Fat-Diet-Induced Mice via Short-Chain Fatty Acids Production and Gut Microbiota Regulation
Source: Foods. 2024 Dec 19;13(24):4113. doi: 10.3390/foods13244113 (PMC11675633; doi:10.3390/foods13244113)

**Figure. S1** UV absorption spectrum of GRP

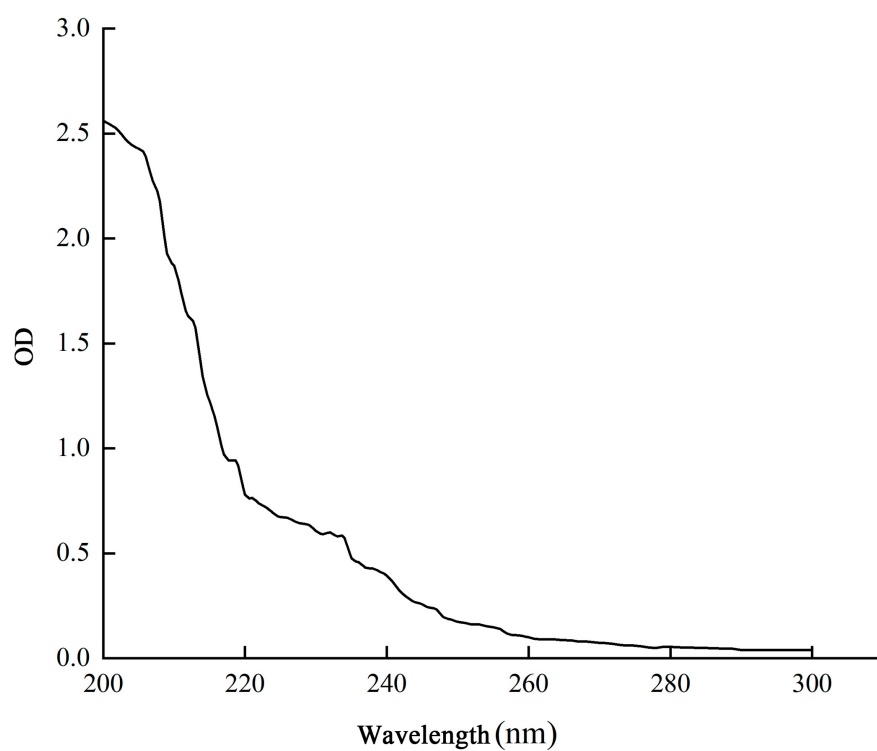

**Figure. S2** GRP IR spectrum

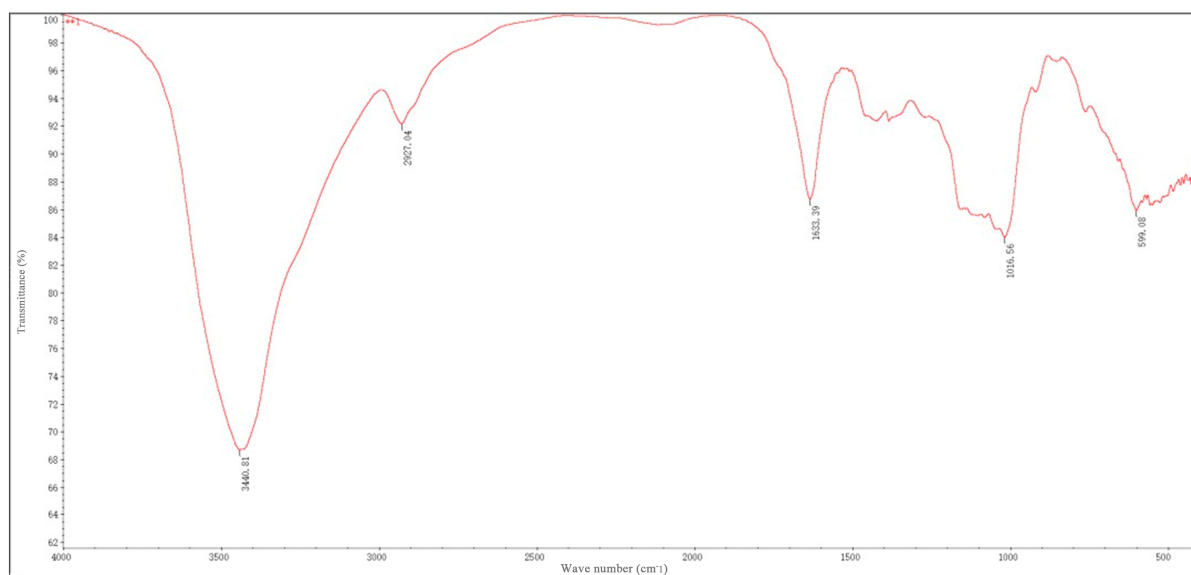

Supplement: Supplementary file 1 [file foods-13-04113-s001.zip › foods-3359015-supplementary.pdf]
